# Prognostic Value of Pre‐Treatment Diffusion Kurtosis Imaging for Progression‐Free Survival Prediction in Advanced Nasopharyngeal Carcinoma

**DOI:** 10.1002/cam4.70883

**Published:** 2025-04-25

**Authors:** Wang Ren, Xiang Zheng, Shizhong Wu, Caixia Wu, Dechun Zheng

**Affiliations:** ^1^ Department of Radiology Clinical Oncology School of Fujian Medical University, Fujian Cancer Hospital Fuzhou People's Republic of China

**Keywords:** diffusion kurtosis imaging, magnetic resonance imaging, nasopharyngeal carcinoma, progression‐free survival

## Abstract

**Purpose:**

This study aimed to evaluate the value of diffusion kurtosis imaging (DKI) for prognostic value for long‐term PFS in nasopharyngeal carcinoma (NPC).

**Methods:**

A cohort of 295 NPC patients underwent pretreatment 3.0T MRI with DKI to derive mean kurtosis (MK), mean diffusion (MD), and apparent diffusion coefficient (ADC). Clinical parameters (Tumor stage, EBV‐DNA, neoadjuvant chemotherapy regimens) were recorded. Follow‐up extended to December 2023. Statistical analyses (R software v4.3.0) included univariate/multivariate Cox regression and Kaplan–Meier survival analysis. A prognostic nomogram integrating key predictors was developed.

**Results:**

Median 10‐year follow‐up revealed 2‐, 5‐, and 10‐year PFS rates of 89%, 79%, and 74%, respectively. Univariate Cox regression analysis demonstrated that T stage, Clinical Stages, NAC regimens, ADC_Group, MK_Group, and MD_Group were significant prognostic factors for PFS in NPC (*p* < 0.05). Multivariate analysis identified Clinical Stage (HR = 2.230, 95% CI 1.44–3.66, *p* < 0.001), NAC (neoadjuvant chemotherapy) regimens (HR = 0.56, 95% CI 0.35–0.90, *p* = 0.017), and MK_Group (HR = 0.52, 95% CI 0.33–0.82, *p* = 0.003) as independent prognostic factors. The MK_Group high exhibited superior survival rates versus MK_Group low (2‐year: 94% vs. 81%; 5‐year: 85% vs. 66%; 10‐year: 79% vs. 64%; all *p* < 0.05). The nomogram combining Clinical Stage, NAC, and MK_Group demonstrated moderate predictive accuracy for 2‐, 5‐, and 10‐year PFS (AUC = 0.736, 0.718, 0.697).

**Conclusion:**

Pretreatment MK serves as a robust noninvasive biomarker for long‐term PFS in NPC. Integration with Clinical Stage and NAC regimens enhances prognostic stratification, supporting personalized therapeutic strategies.

## 1 Introduction

Nasopharyngeal carcinoma (NPC) is a common and aggressive malignancy in southern China characterized by a unique geographic distribution [1]. Despite advancements in screening and treatment, 5%–15% of patients experience local recurrence, and 15%–30% develop metastatic disease after standard treatment [2]. Traditional prognostic tools, such as the TNM staging system and WHO histologic classification, are widely used but offer inadequate prognostic accuracy for NPC patients [3, 4, 5]. These conventional methods often fail to account for the significant clinical variability observed among patients at the same disease stage. Consequently, there is a critical need for more precise prognostic biomarkers to improve patient stratification and therapeutic outcomes.

Diffusion‐weighted imaging (DWI) is widely utilized in clinical practice for routine scans and has demonstrated potential in predicting disease prognosis [6, 7]. DWI assesses tissue water molecule diffusion, presuming Gaussian behavior and mono‐exponential signal decay as *b*‐values increase. However, the prognostic value of DWI is constrained by its inability to accurately represent the complex microenvironment of biological tissues, as initially described by Jensen et al. [8]. Diffusion kurtosis imaging (DKI) [9] utilizes a polynomial model to account for non‐Gaussian water diffusion, necessitating ultra‐high *b*‐values and an adapted image post‐processing method. DKI extends the DWI technique by incorporating kurtosis to measure deviations from Gaussian distribution in water molecule diffusion. By overcoming the theoretical limitations of DWI, DKI offers enhanced prognostic capabilities, making it a valuable imaging biomarker for disease diagnosis, efficacy monitoring, and prognosis prediction.

Recent studies have shown DKI's potential in assessing the prognosis of cancers such as breast cancer [10], pancreatic cancer [11], osteosarcoma [12], glioma [13], and chronic kidney disease [14]. However, few studies have focused on DKI in NPC. Zhao et al. [15] indicated that DKI effectively tracks and monitors acute salivary gland damage in NPC patients. A prospective study demonstrated that mean diffusivity (MD) and mean kurtosis (MK) from DKI could predict the short‐term efficacy of induction chemotherapy in NPC [16]. Previous research suggested that DKI might outperform mono‐exponential DWI in predicting early responses to neoadjuvant chemotherapy in locally advanced NPC [17]. However, these studies had small sample sizes and concentrated on short‐term outcomes. The clinical value of DKI in predicting long‐term outcomes, such as long‐term PFS, in NPC remains unreported.

This prospective study investigates the predictive value of pre‐treatment DKI parameters of primary lesions for PFS in NPC patients. By identifying patients at higher risk of disease progression, clinicians can more effectively customize treatment strategies. Patients with poor prognostic indicators may benefit from more aggressive or innovative treatment modalities and more frequent post‐treatment follow‐up, thereby improving prognostic accuracy and long‐term outcomes.

## 2 Materials and Methods

### 2.1 Patients

**FIGURE 1:**
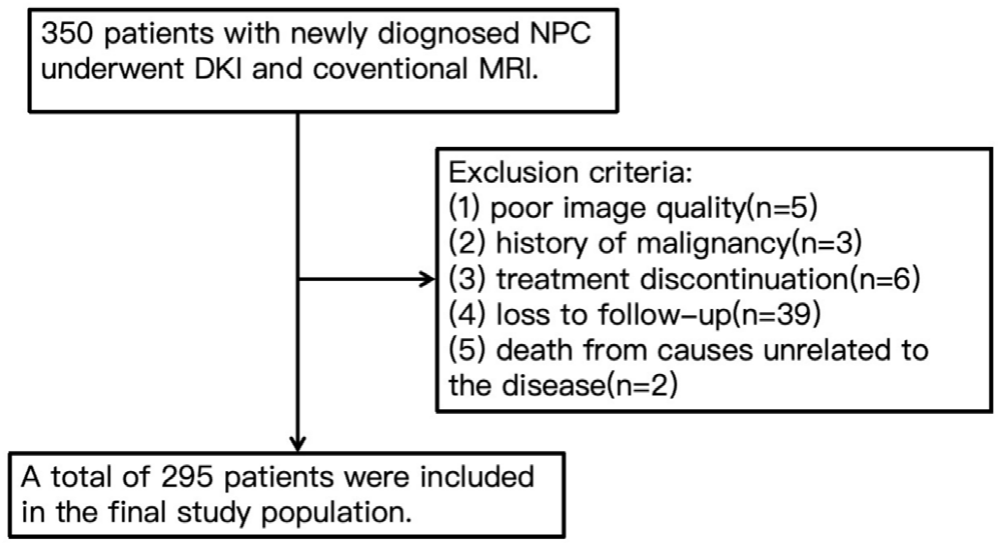
The flowchart of patient inclusion and exclusion.

All experimental procedures were reviewed and approved by the Regional Medical and Health Research Ethics Committee. Informed consent was obtained from each patient after a comprehensive explanation of the study procedures. TNM statuses were determined based on the 8th edition of the AJCC staging system. The inclusion criteria were: (1) histopathologically confirmed nasopharyngeal carcinoma (NPC), (2) clinical stage III to IVa, (3) standard radiotherapy‐based treatment, and (4) complete follow‐up data. The exclusion criteria were: (1) poor image quality affecting DKI measurements, (2) history of malignancy, (3) treatment discontinuation, (4) loss to follow‐up, (5) death from causes unrelated to the disease. Five patients were excluded due to poor image quality, three patients were excluded because of a history of malignancy, six patients discontinued treatment, 39 patients were lost to follow‐up, and two patients died from causes unrelated to the disease. Finally, a total of 295 patients were included in the study population from September 2013 to April 2015. Figure 1 shows the Flowchart of patient inclusion and exclusion.

We collected basic information from the patients, including age, gender, T stage, N stage, clinical stage, histopathological type, and EBV‐DNA level. The EBV DNA levels were measured using venous blood samples. The EBV DNA was detected by real‐time quantitative PCR (qPCR) using specific primers and probes.

### 2.2 Treatment and Follow‐Up

Patients primarily received radiotherapy, supplemented by chemotherapy and other adjuvant treatments such as targeted therapy and immunotherapy. The chemotherapy regimen was cisplatin‐based, administered between 2 and 6 cycles. Radiotherapy was administered using intensity‐modulated radiotherapy (IMRT) with doses of 66–72 Gy for GTVnx, 64–70 Gy for GTVnd, 56–64 Gy for PTV1, and 50–56 Gy for PTV2, across 30–33 fractions. The administration of neoadjuvant chemotherapy involved two (*n* = 236) or three cycles (*n* = 59), determined by clinical staging and individual patient tolerance. Among the participants, 169 (57%) were treated with DDP (cisplatin) at 100 mg/m^2^ (Qilu Pharmaceutical, Shandong, China) on days 1, 2, and 3, combined with 135 mg/m^2^ Paclitaxel (PTX, Hainan Chuntch Pharmaceutical, Hainan, China) on day 1. The remaining 126 (43%) received cisplatin at 100 mg/m^2^ on days 1, 2, and 3, alongside GEM (gemcitabine) at 1000 mg/m^2^ (Jiangsu Hansoh Pharmaceutical, Jiangsu, China) on days 1 and 8.

The endpoint in this research was PFS. PFS duration was determined by measuring the time from initial diagnosis until the occurrence of tumor advancement (including local recurrences or distant spread), death due to NPC, or the final follow‐up appointment. For NPC patients after radical treatment, we follow up with them every 3 months for the first 2 years, every 6 months from years 3 to 5, and annually after 5 years, with follow‐up protocols including clinical examinations such as nasopharyngoscopy and neck palpation, imaging studies such as chest and abdominal CT, abdominal ultrasound or MRI, PET‐CT, and skeletal ECT scans when necessary, laboratory tests such as EBV DNA detection, and symptom monitoring for signs of recurrence or metastasis, while the specific follow‐up plan is adjusted based on the individual patient's condition. For patients who do not return to our hospital for follow‐up examinations, we conduct regular follow‐ups through telephone interviews, letters, or other methods to monitor their condition. Patients were monitored until death or a final review in December 2023.

### 2.3 MRI Imaging and Analysis

All MRI examinations utilized a 3.0 Tesla whole‐body scanner (Achieva TX, Philips Healthcare, Best, Netherlands) with a 16‐channel head and neck coil. Imaging was conducted before any treatment. The standard MRI protocol comprised axial T1‐weighted imaging (T1WI), axial fat‐suppressed fast spin‐echo T2‐weighted imaging (T2WI), and axial mono‐exponential DWI with *b*‐values of 0 and 800 mm^2^/s. Contrast‐enhanced scans were acquired in three planes. The conventional magnetic resonance acquisition parameters were as follows: T2WI STIR, TE = 70 ms, TR = 6896 ms, FOV = 230 × 240 mm, slice thickness/gap = 5/1 mm, number of slices = 36, number of signal averages (NSA) = 2, scan time = 2 min and 4 s.

Diffusion kurtosis imaging DKI utilized a single‐shot spin‐echo echo‐planar imaging (SE‐EPI) sequence with five high *b*‐values (0, 500, 1000, 1500, and 2000 mm^2^/s) in six orthogonal directions. The DKI model was calculated according to the following formula: *S*(*b*) = *S*(0) × exp. (−*b* × MD) + 1/6 × *b* ^2^ × MD^2^ × MK [18, 19]. In this equation, *S*(0), MD, and MK are used as fitting variables, where *S*(*b*) represents the signal at a specific *b* value, and *S*(0) is the baseline signal without the diffusion gradient. MD indicates the diffusivity, while MK describes the peakedness of the water distribution probability. The parameter MD is the diffusion coefficient after correcting for non‐Gaussian effects, whereas MK reflects non‐Gaussian diffusion behavior. As a non‐Gaussian component, MK may illustrate the inhomogeneity of diffusion that cannot be measured using conventional DWI. The acquisition parameters were as follows: TE = 69 ms, TR = 190 ms, FOV = 23 × 24 cm, slice thickness/gap = 5/1 mm, number of slices = 18, IR delay = 240 ms, NSA = 2, SENSE factor = 3, water‐fat shift = minimum, recon voxel size = 1.24 mm, and scan time was 4:57 (min). The diffusion gradients spanned from the skull base to the glottis.

The ADC values were measured on the workstation (Extended MR Work Space 2.6.3.4, Philips Healthcare). DKI parameters, such as MD and mean MK, were measured using IDL 6.3 software (Philips Healthcare). Two radiologists, with 9 and 12 years of experience respectively, independently assessed the DKI parameters of the primary NPC lesions. Both radiologists were blinded to the patients' clinical information and outcomes. The maximum tumor diameter was measured on axial T2WI images of the primary lesion. ROIs were manually delineated along the tumor margins on the DKI images of the slice showing the largest tumor cross‐section, with cystic degeneration and necrotic areas excluded. Measurements were taken twice, and the averages were recorded. Figure 1 shows two groups of representative images of NPC patients with different prognoses.

### 2.4 Statistical Analysis

Statistical analyses were performed using R software (v4.3.0). Descriptive statistics summarized patient characteristics, with intergroup comparisons conducted using independent *t*‐tests, Mann–Whitney *U* tests, Chi‐square tests, or Fisher's exact tests as appropriate. The thresholds for MD, MK, and ADC were determined based on the Maximally Selected Rank Statistics, which maximizes the log‐rank test statistic to identify the optimal cut‐off values. PFS was assessed via the Kaplan–Meier method, and group differences were determined using the log‐rank test. Univariate and multivariate Cox proportional hazards models were constructed, integrating significant variables from the univariate analysis to identify independent prognostic factors. The proportional hazards assumption was validated using Schoenfeld residuals. A Cox regression based nomogram was developed to quantify each factor's contribution to survival outcomes. This nomogram translated the weighted contribution of each factor into a scoring system, predicting patient survival probability by integrating clinical stage and MK values. All statistical tests were two‐sided with a significance level of *p* < 0.05.

## 3 Results

### 3.1 Patient Characteristics and Outcomes

The study included 295 NPC patients, with 228 males (77%) and 67 females (23%), and a median age of 47 years (IQR 40–56 years). According to the eighth edition of the AJCC TNM staging system, 152 patients (52%) were classified as stage III, and 143 patients (48%) were classified as stage IVa. The majority of tumors (86%) were of the undifferentiated type. Table 1 summarizes the distribution of parameters across different groups.

**TABLE 1:** Characteristics of NPC patients.

Characteristic	Patients (%)
Age (47 ± 12)	
< 45 years	128 (43%)
≥ 45 years	167 (57%)
Sex	
Male	228 (77%)
Female	67 (23%)
Pathological type	
Undifferentiated	253 (86%)
Differentiated	82 (14%)
T stage	
T1	23 (7.8%)
T2	52 (18%)
T3	106 (36%)
T4	114 (39%)
N stage	
N1	21 (7.1%)
N2	55 (19%)
N3	182 (62%)
N4	37 (13%)
Clinical stage^a^	
III	152 (52%)
IVa	143 (48%)
EBV DNA (copies/mL)	
< 1500	84 (28%)
≥ 1500	211 (72%)
Long diameter (mm)	
< 38	201 (68%)
≥ 38	94 (32%)
Short diameter (mm)	
< 22	174 (59%)
≥ 22	121 (41%)
NAC regimens	
PTX + DDP	169 (57%)
GEM + DDP	126 (43%)
ADC_Group	
Low	165 (56%)
High	130 (44%)
MD_Group	
Low	262 (89%)
High	33 (11%)
MK_Group	
Low	94 (32%)
High	201 (68%)

Abbreviations: ADC, apparent diffusion coefficient; ADC_Group High, ADC ≥ 0.952 × 10^−3^ mm^2^/s; ADC_Group Low, ADC < 0.952 × 10^−3^ mm^2^/s; DDP, cisplatin; EB‐DNA, Epstein–Barr Virus DNA; GEM, gemcitabine; MD, mean diffusion; MD_Group High, MD ≥ 1.53 × 10^−3^ mm^2^/s; MD_Group Low, MD < 1.53 × 10^−3^ mm^2^/s; MK, mean kurtosis; MK_Group High, MK ≥ 1.016; MK_Group Low, MK < 1.016; NAC, neoadjuvant chemotherapy; PTX, paclitaxel.

^a^ TNM stage was classified according to the AJCC (American Joint Committee on Cancer) eighth edition.

During the follow‐up, a total of 79 (27%) patients experienced disease progression, including 12 deaths, 24 cases of local recurrence, 9 cases of regional lymph node metastasis, and 34 cases of distant metastasis. Among the distant metastasis cases, the most common sites were bone, lung, liver, and distant lymph nodes. Specifically, there was 1 case of isolated brain metastasis and 1 case of concurrent metastasis to the liver, spleen, bone, and distant lymph nodes. Additionally, distant metastases often involved multiple organs simultaneously, with 2 cases of isolated lung metastasis, 9 cases of isolated bone metastasis, and 3 cases of isolated liver metastasis. The PFS rates at 2, 5, and 10 years were 89% (95% CI: 86%–93%), 79% (95% CI: 74%–83%), and 74% (95% CI: 69%–79%), respectively.

### 3.2 Correlation Analysis

A strong negative correlation was observed between MD and MK (*r* = −0.461, *p* < 0.001). MD exhibited positive correlations with short diameter (*r* = 0.117, *p* = 0.046) and ADC (*r* = 0.275, *p* < 0.001). MK showed negative correlations with long diameter (*r* = −0.102, *p* = 0.037), short diameter (*r* = −0.122, *p* = 0.037), and ADC (*r* = −0.157, *p* = 0.007). Long and short diameters were highly positively correlated (*r* = 0.882, *p* < 0.001). A significant negative association was found between EB‐DNA and ADC (*r* = −0.167, *p* = 0.004). Table 2 summarizes the linear correlations between the continuous variables.

**TABLE 2:** Distribution of correlation coefficients and *p* values for continuous variables.

	MD	MK	Long diameter	Short diameter	EB‐DNA	ADC
MD	1.000	−0.461 (*p* < 0.001)	0.098 (*p* = 0.092)	0.117 (*p* = 0.046)	−0.064 (*p* = 0.274)	0.275 (*p* < 0.001)
MK	−0.461 (*p* < 0.001)	1.000	−0.102 (*p* = 0.037)	−0.122 (*p* = 0.037)	0.045 (*p* = 0.444)	−0.157 (*p* = 0.007)
Long diameter	0.098 (*p* = 0.092)	−0.102 (*p* = 0.037)	1.000	0.882 (*p* < 0.001)	0.019 (*p* = 0.74)	0.023 (*p* = 0.694)
Short diameter	0.117 (*p* = 0.046)	−0.122 (*p* = 0.037)	0.882 (*p* < 0.001)	1.000	0.014 (*p* = 0.805)	0.050 (*p* = 0.394)
EB‐DNA	−0.064 (*p* = 0.274)	0.045 (*p* = 0.444)	0.019 (*p* = 0.74)	0.014 (*p* = 0.805)	1.000	−0.167 (*p* = 0.004)
ADC	0.275 (*p* < 0.001)	−0.157 (*p* = 0.007)	0.023 (*p* = 0.694)	0.050 (*p* = 0.394)	−0.167 (*p* = 0.004)	1.000

Abbreviations: ADC, apparent diffusion coefficient; EB‐DNA, Epstein–Barr Virus DNA; MD, mean diffusion; MK, mean kurtosis.

### 3.3 Cox Regression Analysis (Univariate and Multivariate)

The thresholds were set at 1.530 × 10^−3^ mm^2^/s for MD, 1.016 for MK, and 0.952 × 10^−3^ mm^2^/s for ADC according to Maximally Selected Rank Statistics. Diffusion parameters were categorized as follows: ADC_Group Low (ADC < 0.952 × 10^−3^ mm^2^/s) and ADC_Group High (ADC ≥ 0.952 × 10^−3^ mm^2^/s), MD_Group Low (MD < 1.53 × 10^−3^ mm^2^/s) and MD_Group High (MD ≥ 1.53 × 10^−3^ mm^2^/s), MK_Group Low (MK < 1.016) and MK_Group High (MK ≥ 1.016). Univariate Cox regression analysis demonstrated that T stage (HR = 1.39, 95% CI 1.07–1.81, *p* = 0.014), Clinical Stages (HR = 2.35, 95% CI 1.47–3.74, *p* < 0.001), NAC regimens (HR = 0.57, 95% CI 0.35–0.91, *p* = 0.019), ADC_Group (HR = 0.62, 95% CI 0.39–0.99, *p* = 0.044), MK_Group (HR = 0.53, 95% CI 0.34–0.83, *p* = 0.005), and MD_Group (HR = 1.83, 95% CI 1.03–3.26, *p* = 0.040) were significant prognostic factors for PFS in NPC. Gender, Pathological type, N stage, Age, EBV‐DNA, Long Diameter, Short Diameter were not significant prognostic factors for PFS in NPC (*p* > 0.05). Multivariate Cox regression analysis further identified Clinical Stages (HR = 2.230, 95% CI 1.44–3.66, *p* < 0.001), NAC regimens (HR = 0.56, 95% CI 0.35–0.90, *p* = 0.017), and MK_Group (HR = 0.52, 95% CI 0.33–0.82, *p* = 0.003) as independent prognostic factors for PFS in NPC. ADC_Group, MD_Group, T stage were not independent prognostic factors for PFS in NPC (*p* > 0.05). Table 3 displays the findings from both univariate and multivariate Cox regression analyses.

**TABLE 3:** Univariate and multivariate Cox regression analysis of clinical indicators and DKI parameters with PFS in NPC patients.

Parameter	Univariate Cox regression analysis		Multivariate Cox regression analysis	
	HR (95% CI)	*p*	HR (95% CI)	*p*
Gender				
F^a^				
M	0.93 (0.55–1.56)	0.780	—	—
Pathological type^b^				
Undifferentiated^a^				
Differentiated	0.74 (0.37–1.48)	0.391	—	—
T stage				
1^a^				
2–4	1.39 (1.07–1.81)	0.014	—	—
N stage				
0^a^				
1–3	1.21 (0.88–1.65)	0.235	—	—
Clinical stages				
Stage III^a^				
Stage IVa	2.35 (1.47–3.74)	< 0.001	2.230 (1.44–3.66)	< 0.001
Age				
< 45 years^a^				
≥ 45 years	0.88 (0.56–1.37)	0.564	—	—
EBV‐DNA (copies/mL)				
< 1500^a^				
≥ 1500	1.37 (0.86–2.18)	0.183	—	—
Long diameter (mm)				
< 38^a^				
≥ 38	1.14 (0.72–1.81)	0.584	—	—
Short diameter (mm)				
< 22^a^				
≥ 22	1.18 (0.76–1.84)	0.470	—	—
NAC regimens				
PTX + DDP^a^				
GEM + DDP	0.57 (0.35–0.91)	0.019	0.56 (0.35–0.90)	0.017
ADC_Group				
Low^a^				
High	0.62 (0.39–0.99)	0.044	—	—
MK_Group				
Low^a^				
High	0.53 (0.34–0.83)	0.005	0.52 (0.33–0.82)	0.003
MD_Group				
Low^a^				
High	1.83 (1.03–3.26)	0.040	—	—

Abbreviations: ADC, apparent diffusion coefficient; CI, confidence interval; HR, hazard ratio; MD, mean diffusion; MK, mean kurtosis.

^a^ Used as reference.

^b^ WHO classification.

### 3.4 Survival Analysis

**FIGURE 2:**
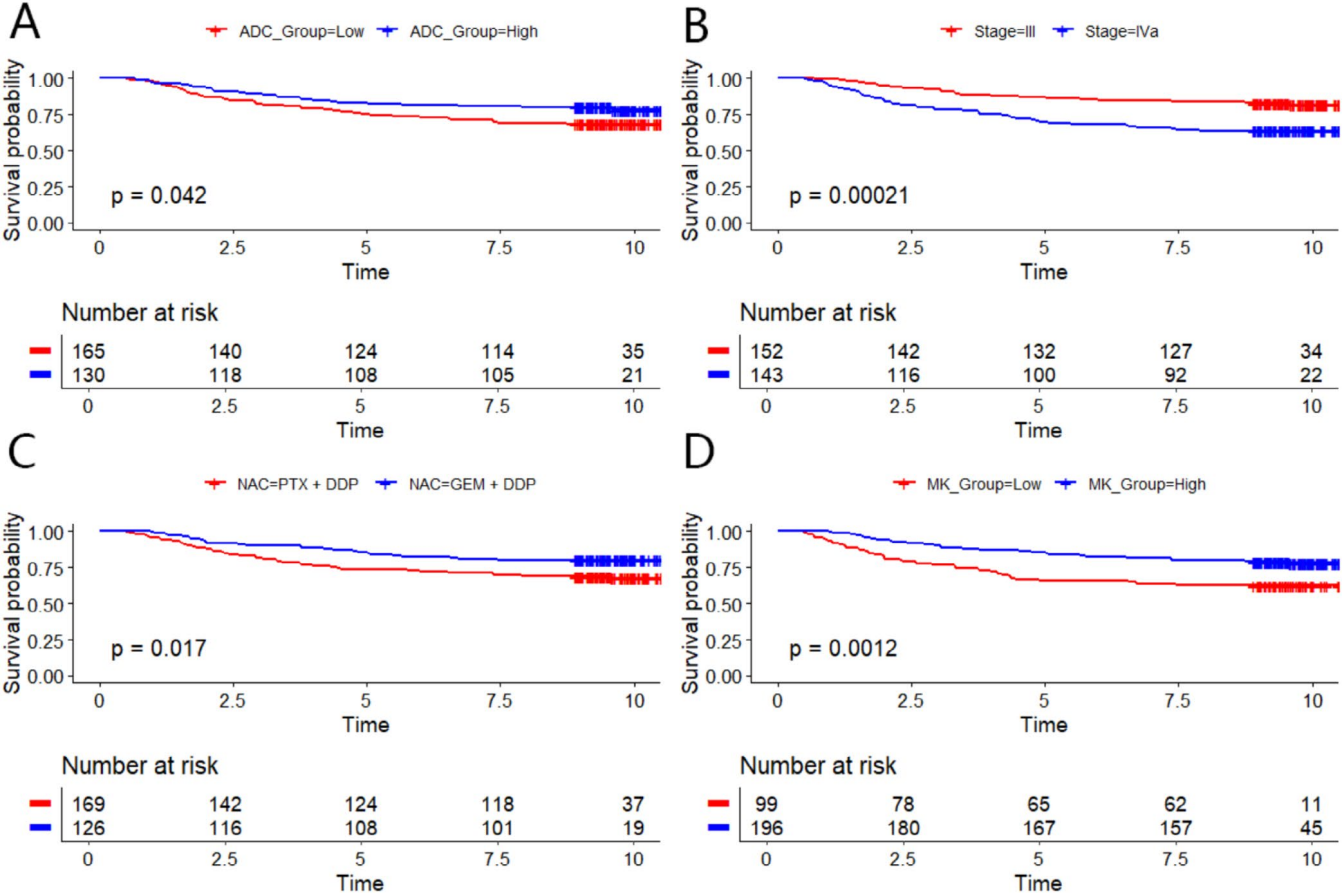
Impact of different risk factors on the survival of nasopharyngeal carcinoma patients. (A–D) Represent the PFS survival curves for patients stratified by ADC_Group, Clinical Stage, NAC regimen, and MK_Group, respectively. (A) Kaplan–Meier survival curves comparing ADC_Group Low (ADC < 0.952 × 10^−3^ mm^2^/s, red line) and ADC_Group High (ADC ≥ 0.952 × 10^−3^ mm^2^/s, blue line). Patients with higher ADC values exhibited significantly better PFS (*p* = 0.042). (B) Kaplan–Meier survival curves comparing stage III (red line) and stage IVa (blue line). Patients in the clinical stage IVa group had a significantly lower survival rate than those in the stage III group (*p* = 0.00021). (C) Kaplan–Meier survival curves comparing different NAC regimens. Patients treated with the PTX + DDP (paclitaxel + cisplatin) neoadjuvant chemotherapy regimen (red line) demonstrated significantly worse progression‐free survival (PFS) outcomes compared to those receiving the GEM + DDP (gemcitabine + cisplatin) regimen (blue line) (*p* = 0.0017). (D) Kaplan–Meier survival curves comparing MK_Group Low (MK < 1.016, red line) and MK_Group High (MK ≥ 1.016 × 10^−3^ mm^2^/s, blue line). Patients with lower MK values exhibited significantly worse PFS (*p* = 0.0012).

Figure 2 displays the Kaplan–Meier survival curves for various patient groups. Panels A–D represent the PFS survival curves for patients stratified by ADC_Group, clinical stage, NAC regimen, and MK_Group, respectively. Panel A: Kaplan–Meier survival curves comparing ADC_Group Low (ADC < 0.952 × 10^−3^ mm^2^/s, red line) and ADC_Group High (ADC ≥ 0.952 × 10^−3^ mm^2^/s, blue line). Patients with higher ADC values exhibited significantly better PFS (*p* = 0.042). Panel B: Kaplan–Meier survival curves comparing stage III (red line) and stage IVa (blue line). Patients in the clinical stage IVa group had a significantly lower survival rate than those in the stage III group (*p* = 0.00021). Panel C: Kaplan–Meier survival curves comparing different NAC regimens. Patients treated with the PTX + DDP neoadjuvant chemotherapy regimen (red line) demonstrated significantly worse progression‐free survival (PFS) outcomes compared to those receiving the GEM + DDP regimen (blue line) (*p* = 0.0017). Panel D: Kaplan–Meier survival curves comparing MK_Group Low (MK < 1.016, red line) and MK_Group High (MK ≥ 1.016 × 10^−3^ mm^2^/s, blue line). Patients with lower MK values exhibited significantly worse PFS (*p* = 0.0012).

**FIGURE 3:**
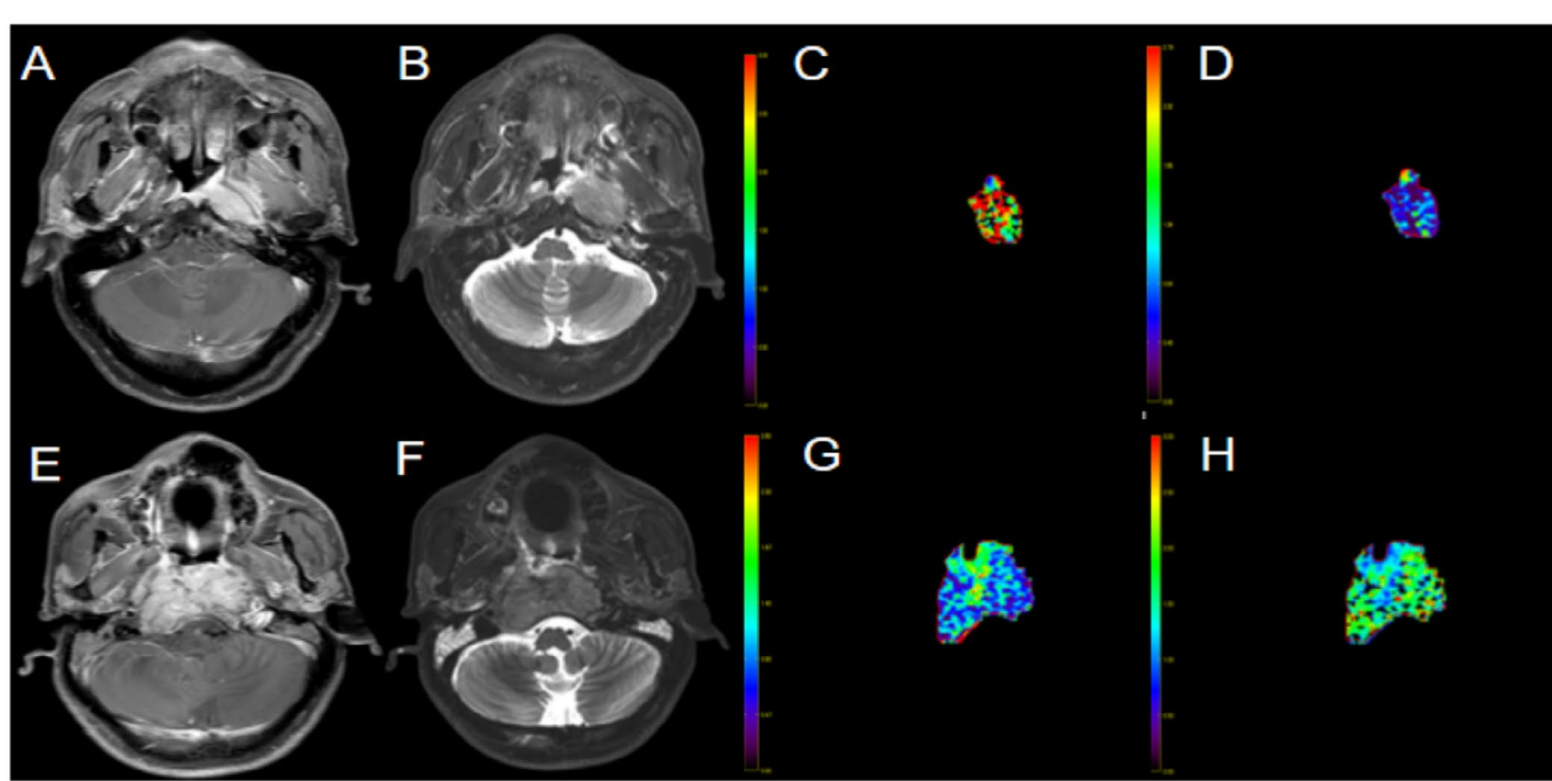
Representative images of two groups of nasopharyngeal carcinoma (NPC) patients with different prognoses. (A, E) Display T1WI fat‐suppression enhanced images, (B, F) show T2WI fat‐suppression images, (C, G) exhibit MD parametric maps, while (D, H) exhibit MK parametric maps. The upper row of images belongs to a 51‐year‐old male patient diagnosed with non‐keratinizing undifferentiated nasopharyngeal carcinoma, staged as T4N1M0 IVa. Before treatment, the MD value was relatively low (0.7395 × 10^−3^ mm^2^/s), while the MK value was elevated (1.736). This patient demonstrated a good prognosis with no recurrence, metastasis, or disease‐related death after treatment. The lower row of images represents a 52‐year‐old male patient with non‐keratinizing undifferentiated NPC, staged as T4N2M0 IVa. Prior to treatment, the MD value was higher (1.093 × 10^−3^ mm^2^/s), while the MK value was lower (1.028). However, 15 months after treatment, follow‐up MRI and biopsy confirmed local recurrence.

Table 4 presents the PFS rates at 2, 5, and 10 years for various patient subgroups. The overall survival rate for NPC patients is 89% at 2 years (95% CI: 86%–93%), 79% at 5 years (95% CI: 74%–83%), and 74% at 10 years (95% CI: 69%–79%). Patients receiving the GEM + DDP regimen exhibit better survival rates than those on the PTX + DDP regimen, with a 5‐year survival rate of 86% versus 73%, and a 10‐year survival rate of 80% versus 67%. The differences are statistically significant (*p* = 0.007, *p* = 0.022). The low ADC group shows significantly lower survival rates compared to the high ADC group, particularly at the 10‐year mark (68% vs. 78%, *p* = 0.022). These findings suggest that ADC levels may influence long‐term survival, with lower ADC levels being associated with worse outcomes. Survival rates for the high MK group are significantly higher than for the low MK group at all time points (2 years: 94% vs. 81%; 5 years: 85% vs. 66%; 10 years: 79% vs. 64%). Statistically significant differences are observed at each time point (*p* = 0.004, 0.001, 0.013), indicating that MK group status is an important prognostic factor for long‐term survival in NPC patients (Figure 3). Patients with clinical stage III NPC exhibit significantly better survival rates than those with stage IVa, particularly at the 5‐year (87% vs. 70%) and 10‐year (84% vs. 64%) time points. The differences are highly significant (*p* < 0.001), highlighting the critical role of clinical staging in predicting long‐term survival in NPC patients. The survival rates for the low MD group are significantly higher than those for the high MD group, particularly at the 5‐year (81% vs. 64%) and 10‐year (76% vs. 58%) marks, with a *p*‐value of 0.046 at 10 years. This indicates that the MD group classification may have a significant impact on long‐term survival, with the high MD group associated with poorer outcomes. The age, however, showed minimal impact on survival, with no significant differences observed.

**TABLE 4:** Survival rates and comparative analysis among different subgroups of NPC patients.

	2 years (95% CI)	5 years (95% CI)	10 years (95% CI)
Overall	89% (86%, 93%)	79% (74%, 83%)	74% (69%, 79%)
Age			
< 45 years	89% (84%, 95%)	75% (68%, 83%)	73% (65%, 81%)
≥ 45 years	90% (85%, 95%)	81% (76%, 88%)	75% (69%, 82%)
*p*	0.834	0.186	0.567
MD_Group			
Low	90% (86%, 93%)	81% (76%, 85%)	76% (71%, 81%)
High	88% (77%, 100%)	64% (49%, 82%)	58% (43%, 77%)
*p*	0.762	0.053	0.046
NAC regimens			
PTX + DDP	88% (83%, 93%)	73% (67%, 80%)	67% (61%, 75%)
GEM + DDP	92% (87%, 97%)	86% (80%, 92%)	80% (73%, 87%)
*p*	0.199	0.007	0.022
ADC_Group			
Low	87% (82%, 92%)	75% (69%, 82%)	68% (62%, 76%)
High	93% (89%, 98%)	83% (77%, 90%)	78% (71%, 86%)
*p*	0.064	0.092	0.022
MK_Group			
Low	81% (73%, 89%)	66% (57%, 76%)	64% (55%, 74%)
High	94% (90%, 97%)	85% (80%, 90%)	79% (73%, 84%)
*p*	0.004	0.001	0.013
Clinical stage^a^			
III	95% (91%, 98%)	87% (82%, 92%)	84% (78%, 90%)
IVa	84% (78%, 90%)	70% (63%, 78%)	64% (56%, 72%)
*p*	0.002	< 0.001	< 0.001

Abbreviations: ADC, apparent diffusion coefficient; DDP, cisplatin; GEM, gemcitabine; MD, mean diffusion; MK, mean kurtosis; NAC, neoadjuvant chemotherapy; PTX, taxol.

^a^ TNM stage was classified according to the AJCC (American Joint Committee on Cancer) eighth edition.

### 3.5 Prognostic Model and Validation

**FIGURE 4:**
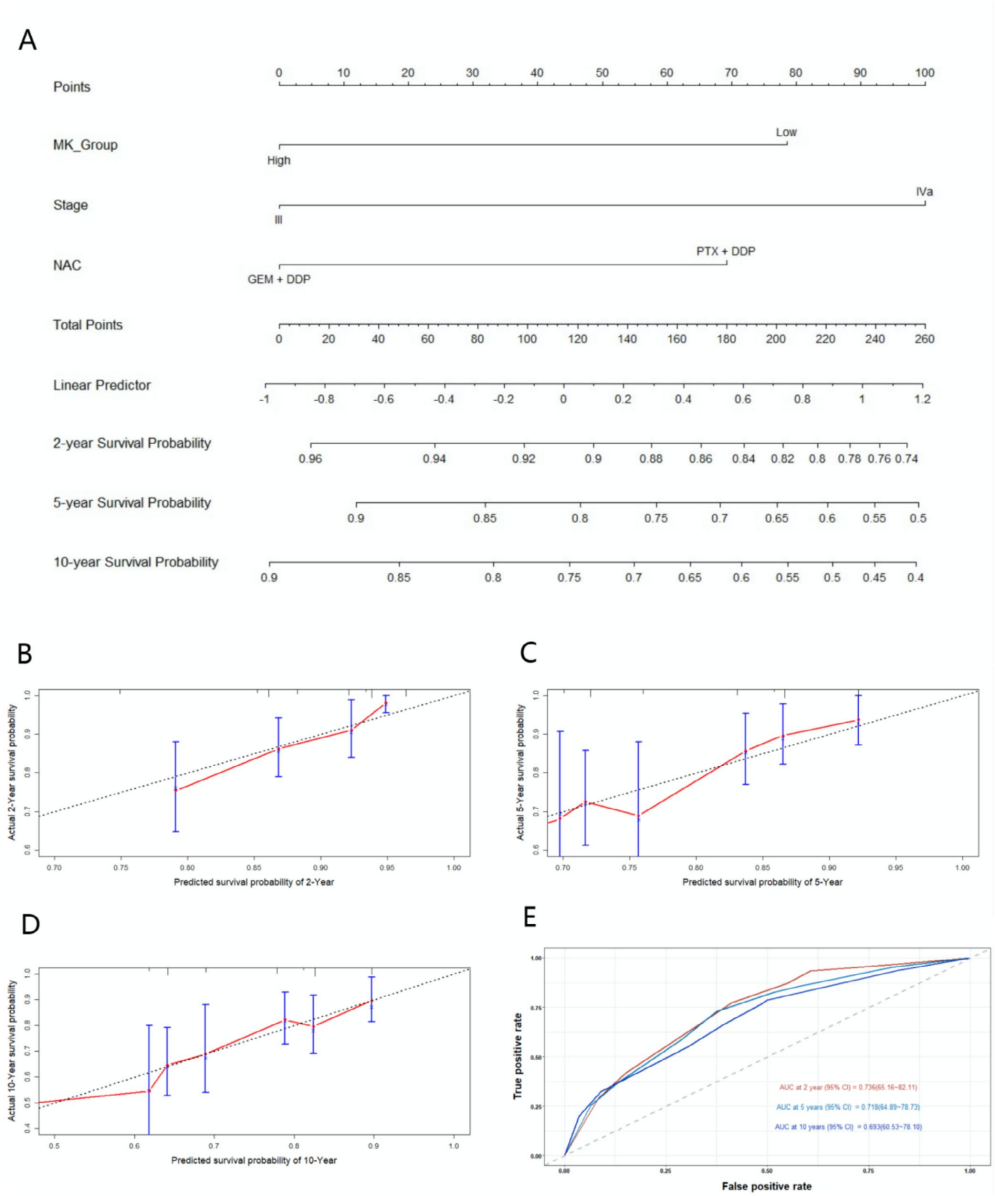
Nomogram and calibration curves. (A) Shows a nomogram for survival probability prediction. This panel illustrates the nomogram used to predict the 2, 5, and 10‐year survival probabilities of NPC patients based on MK_Group, Clinical Stage, and NAC regimen. (B–D) Present calibration plots for the 2, 5, and 10‐year survival probabilities, respectively. The dotted line represents perfect calibration and the red line shows the observed performance of the model. The proximity of the red line to the dotted line indicates good calibration. These plots show that the model performs well in predicting survival probabilities at different time points. (E) Displays the receiver operating characteristic (ROC) curve, which is used to evaluate the discrimination ability of the survival prediction model. The AUC values for the survival prediction model are 0.746 at 2 years, 0.718 at 5 years, and 0.693 at 10 years.

Figure 4A shows a nomogram for survival probability prediction. This panel illustrates the nomogram used to predict the 2‐, 5‐, and 10‐year survival probabilities of NPC patients based on M_Group, Clinical Stage, and NAC Regimen. Each factor is assigned a certain number of points (depicted at the top of the nomogram). For example, patients in the high MK_Group are assigned higher points, while patients in stage IVa receive a higher point total compared to those in stage III. Additionally, GEM + DDP NAC regimen receives higher points than PTX + DDP. The total points (obtained by summing the points from all factors) are used to predict the survival probabilities. The linear predictor, derived from the total points, is then mapped to the survival probabilities for 2, 5, and 10 years. Figure 4B–D presents calibration plots for the 2‐, 5‐, and 10‐year survival probabilities, respectively. These plots assess the accuracy of the predicted survival probabilities against the actual observed survival outcomes. The dotted line represents perfect calibration (where predicted survival equals actual survival), and the red line shows the observed performance of the model. The proximity of the red line to the dotted line indicates good calibration. These plots show that the model performs well in predicting survival probabilities at different time points. Figure 4E displays the Receiver Operating Characteristic (ROC) curve, which is used to evaluate the discrimination ability of the survival prediction model. The AUC values for the survival prediction model are 0.736 (95% CI 65.16–82.11) at 2 years, 0.718 (95% CI 64.89–78.73) at 5 years, and 0.693 (95% CI 60.53–78.10) at 10 years, indicating strong discriminatory ability at all time points.

## 4 Discussion

Early prediction of NPC efficacy aids clinicians in stratifying patients based on prognostic risk. For patients identified with adverse prognostic factors, more aggressive treatment approaches may be implemented, such as increasing radiation dosage, extending chemotherapy duration, and utilizing combined targeted or immunotherapy. Additionally, an increase in follow‐up frequency post‐treatment can improve patient prognosis. This study assesses the prognostic significance of DKI parameters for predicting PFS in NPC patients. Our findings suggest that Clinical Stages, NAC regimens, and MK_Group were independent prognostic factors for PFS of NPC. Patients with ADC_Group high, Clinical Stage III, and MK_Group high exhibited significantly better PFS. We developed a visual nomogram based on independent prognostic factors identified through clinical and DKI analysis, facilitating reference for both clinicians and patients.

DKI, a mathematical model introduced by Jensen et al. [9], provides an enhanced framework for describing non‐Gaussian characteristics in biological tissues and the deviations of true diffusion from a Gaussian distribution. The two parameters derived from DKI (MD and MK) offer improved depiction of changes in tissue microstructure, particularly in tumors. The present study noted that higher pre‐treatment MK values are associated with better PFS rates, which may suggest increased irregularity and heterogeneity in the tissue microstructure. Many studies have evaluated the efficacy of DKI parameters compared to mono‐exponential DWI parameters in predicting the malignancy of various tumors throughout the body [11, 20, 21]. Although the value of DKI in predicting the long‐term efficacy of NPC has not been reported previously, some published studies have indicated that pre‐treatment *K* values in RG (Responder Group) differ from those in NRG (Non‐Responder Group) for other types of cancer [22, 23]. In a study on the predictive value of pre‐treatment DKI for induction chemotherapy in locally advanced NPC, Zhao et al. [16] found that the MD values in the responding group were significantly lower than those in the non‐responding group (*p* = 0.001). Conversely, the MK values in the responding group were significantly higher than those in the non‐responding group (*p* = 0.017). These findings are consistent with the results of the current study, suggesting that patients with higher MK values and lower MD values may exhibit greater sensitivity to induction chemotherapy, resulting in better prognoses.

However, some studies on other tumors showed that the MK value was lower in the effective group, which may be due to the treatment methods of different tumors [24]. NPC is a malignant tumor mainly treated by radiotherapy. Patients who are more sensitive to radiotherapy tend to have a better prognosis. Tumors with higher MK values indicate a greater deviation from the Gaussian distribution, suggesting an increased number of factors influencing water diffusion. Specifically, elevated MK values reflect higher cell density, increased cellular activity, and a more crowded intracellular environment [18, 25], which may be caused by a higher proliferation rate in tumor cells [26]. Tumors with higher cell proliferation tend to be more sensitive to radiotherapy, which theoretically explains why NPC patients with a higher MK value have a better prognosis. Zheng et al. [25] observed in their study on NPC xenografts in nude mice that as the number of radiation treatments increased, MD values rose while MK values decreased. MK values decreased significantly in the radiation‐sensitive group, whereas no notable change was observed in the low‐sensitivity group. This suggests that MK values can predict the radiation sensitivity of NPC. Patients with higher MK value are more sensitive to radiotherapy and have a better prognosis.

Compared to ADC values obtained from traditional mono‐exponential models, MD values provide a more accurate reflection of the extent of restricted diffusion within biological tissues by utilizing a model that accounts for the non‐Gaussian distribution of water molecule diffusion. Jansen et al. [26] found that the kurtosis model demonstrated a significantly better fit to the experimental data points compared to the mono‐exponential diffusion model. This approach holds promise for more precise predictions of disease prognosis. Furthermore, there is a clear negative correlation between MD and MK values, which has been similarly confirmed in previous studies [17]. As we all know, the MD value is actually the corrected ADC value. Previous studies have confirmed that the lower the ADC value, the better the prognosis of locally advanced head and neck cancer, reflecting the consistency of the results of this study and historical studies [27]. DKI's superior accuracy in representing the tumor microenvironment over traditional DWI is well confirmed across various cancers, such as breast cancer [10], pancreatic cancer [11], and gliomas [21, 28].

By capturing non‐Gaussian diffusion behavior, DKI provides critical insights into tumor complexity and heterogeneity, which are essential for predicting patient outcomes and guiding treatment strategies. For patients with poor DKI‐derived prognosis, we recommend intensifying induction chemotherapy, incorporating targeted or immunotherapy, and optimizing radiotherapy doses or courses. Additionally, closer post‐treatment surveillance should be advised for high‐risk patients. Multidisciplinary collaboration is essential to integrate DKI insights into treatment planning, ultimately improving patient outcomes.

Despite these encouraging results, some challenges remain in implementing DKI in clinical practice. One of the main challenges is the need for standardized imaging protocols and data analysis methods [29]. Variability in imaging protocols, scanner settings, and post‐processing techniques can lead to inconsistent results, limiting the generalizability of findings across different institutions and patient populations [30]. To address this issue, efforts should be made to develop consensus guidelines for DKI imaging in NPC and other cancers, ensuring that the technique can be reliably integrated into routine clinical practice. Additionally, we acknowledge the growing interest in artificial intelligence (AI) and radiomics, which have demonstrated significant potential in tumor prognosis prediction [31, 32]. However, whether AI can extract more effective information from DKI images for predicting NPC prognosis remains an area requiring further investigation.

This study has several limitations. First, due to the requirement for a certain tumor volume for accurate DKI measurements, we only included patients with advanced‐stage NPC. The applicability of DKI in early stage NPC and other cancer types remains to be further explored. Second, although our cohort of 295 patients is adequate for primary analyses, the sample size may limit the statistical power of subgroup analyses, particularly for detecting smaller effect sizes. Future studies with larger, multicenter cohorts are needed to validate and extend our findings. Furthermore, although this study was prospective and all patients were scanned on the same machine to minimize bias from different field strengths and scanner models, the imaging technology at the time of the study was less mature. The *b*‐values and imaging directions in DKI were insufficient, and other DKI parameters, such as radial kurtosis (RK) and axial kurtosis (AK), were not collected. These parameters could provide diverse perspectives, yielding more comprehensive information for tumor characterization and prognosis prediction. Future research should investigate the role of these parameters across different cancer types and evaluate their potential for combined use with MK in enhancing prognostic predictions.

## 5 Conclusion

In this prospective study, we investigated the value of DKI parameters in predicting PFS in 295 untreated NPC patients. Studies have confirmed that the parameter MK generated by DKI is an independent risk factor for the prognosis of NPC. Judging from the survival curve, MK is indeed expected to provide a valuable imaging marker in the prognosis stratification of NPC. As future research advances and technology continues to mature, DKI is poised to play a significant role in clinical oncology, providing new tools and methods for improving the long‐term prognosis of patients with various malignancies. We believe that through further research and standardized global application, DKI will become an integral part of enhancing cancer diagnosis and treatment outcomes, driving the development of personalized medicine, and ultimately improving patient quality of life and survival rates.

## Author Contributions

Wang Ren: conceptualization, data curation, formal analysis, project administration, writing – original draft. Xiang Zheng: MRI image analysis and quantitative parameter measurement. Shizhong Wu: patient data acquisition and organization. Caixia Wu: patient follow‐up and clinical outcome tracking. Dechun Zheng: study design and methodology. All authors had full access to all study data and take responsibility for the integrity of the data and the accuracy of the analysis. Additionally, all authors critically reviewed the manuscript and approved the final version for publication.

## Ethics Statement

The study design was approved by the Medical Ethics Committee of Fujian Cancer Hospital and conducted in adherence to the Declaration of Helsinki.

## Conflicts of Interest

The authors declare no conflicts of interest.

## Data Availability

The additional data collected in this study are available from the corresponding authors upon reasonable request.
